# Biodegradable magnesium alloy with eddy thermal effect for effective and accurate magnetic hyperthermia ablation of tumors

**DOI:** 10.1093/nsr/nwaa122

**Published:** 2020-06-10

**Authors:** Nailin Yang, Fei Gong, Liang Cheng, Huali Lei, Wei Li, Zongbin Sun, Caifang Ni, Zhanhui Wang, Zhuang Liu

**Affiliations:** Institute of Functional Nano & Soft Materials (FUNSOM), Jiangsu Key Laboratory for Carbon-Based Functional Materials and Devices, Soochow University, Suzhou 215123, China; Institute of Functional Nano & Soft Materials (FUNSOM), Jiangsu Key Laboratory for Carbon-Based Functional Materials and Devices, Soochow University, Suzhou 215123, China; Institute of Functional Nano & Soft Materials (FUNSOM), Jiangsu Key Laboratory for Carbon-Based Functional Materials and Devices, Soochow University, Suzhou 215123, China; Institute of Functional Nano & Soft Materials (FUNSOM), Jiangsu Key Laboratory for Carbon-Based Functional Materials and Devices, Soochow University, Suzhou 215123, China; Department of Vascular Surgery and Interventional Radiology, First Affiliated Hospital of Soochow University, Suzhou 215006, China; Luoyang Central Hospital affiliated to Zhengzhou University, Luoyang 471000, China; Department of Vascular Surgery and Interventional Radiology, First Affiliated Hospital of Soochow University, Suzhou 215006, China; Luoyang Central Hospital affiliated to Zhengzhou University, Luoyang 471000, China; Institute of Functional Nano & Soft Materials (FUNSOM), Jiangsu Key Laboratory for Carbon-Based Functional Materials and Devices, Soochow University, Suzhou 215123, China

**Keywords:** magnesium alloy, eddy thermal effect, alternating magnetic field, hyperthermia tumor ablation, biodegradation

## Abstract

Magnetic hyperthermia therapy (MHT) is able to ablate tumors using an alternating magnetic field (AMF) to heat up magnetocaloric agents (e.g. magnetic nanoparticles) administered into the tumors. For clinical applications, there is still a demand to find new magnetocaloric agents with strong AMF-induced heating performance and excellent biocompatibility. As a kind of biocompatible and biodegradable material, magnesium (Mg) and its alloys have been extensively used in the clinic as an implant metal. Herein, we discovered that the eddy thermal effect of the magnesium alloy (MgA) could be employed for MHT to effectively ablate tumors. Under low-field-intensity AMFs, MgA rods could be rapidly heated, resulting in a temperature increase in nearby tissues. Such AMF-induced eddy thermal heating of MgA could not only be used to kill tumor cells *in vitro*, but also be employed for effective and accurate ablation of tumors *in vivo*. In addition to killing tumors in mice, we further demonstrated that VX_2_ tumors of much larger sizes growing in rabbits after implantation of MgA rods could also be eliminated after exposure to an AMF, illustrating the ability of MgA-based MHT to kill large-sized tumors. Moreover, the implanted MgA rods showed excellent biocompatibility and ∼20% of their mass was degraded within three months. Our work thus discovered for the first time that non-magnetic biodegradable MgA, an extensively used implant metal in clinic, could be used for effective magnetic thermal ablation of tumors under a low-field-intensity AMF. Such a strategy could be readily translated into clinical use.

## Teaser

A new strategy using non-magnetic and biodegradable Mg alloy under a low-field-intensity alternating magnetic field was developed for accurate and effective tumor treatment in a minimally invasive manner, applicable even for deep-set and large tumors.

## INTRODUCTION

Magnetic hyperthermia therapy (MHT) has attracted much attention since the 1980s as a non-invasive local treatment strategy to destruct tumors under an external alternating magnetic field (AMF) [1–4]. During MHT, tumors are often administered with magnetocaloric agents [5], unusually magnetic nanoparticles (NPs) [6,7], which are able to generate heat under a strong AMF, consequently resulting in the death of tumor cells [8–11]. Compared to other types of clinically used local hyperthermia therapies induced by laser [12–15], radiofrequency [16,17], microwave [18], or high-intensity focused ultrasound [19,20], the magnetic field used in MHT has no tissue penetration limits, and does not cause any heating effect to tissues in the absence of magnetocaloric agents, promising an accurate ablation of deep-set internal tumors even those of large size [21,22]. Despite the advantages of MHT for non-invasive local tumor treatment, the clinical use of MHT remains rare [23,24]. One reason that hampers the clinical application of MHT is the magnetocaloric agent [25]. In addition to safety concerns, the currently used magnetic NPs (e.g. iron oxide) can only be effectively heated under rather strong AMFs (H_appl_ × f_appl_ > 5 × 10^9^ A · m^−1^ · s^−1^) [26,27]. There is still a need to develop safer and more effective magnetocaloric agents that could be implanted into tumors for MHT tumor ablation.

In addition to magnetic NPs, whose AMF-induced heating mechanism is mainly due to the heating power of relaxation loss [28,29], bulk conductors such as metals can also be heated under an AMF by the eddy current effect, in which an induced current is generated when a bulk conductor is placed in an AMF [30,31]. When the resistivity of the conductor is small (e.g. for metals), the AMF-induced eddy current would be quite strong and the generated heat due to the eddy thermal effect would be very large [32]. Therefore, it would be interesting to use the eddy thermal effect of bulk metal for tumor ablation.

**Scheme 1.**
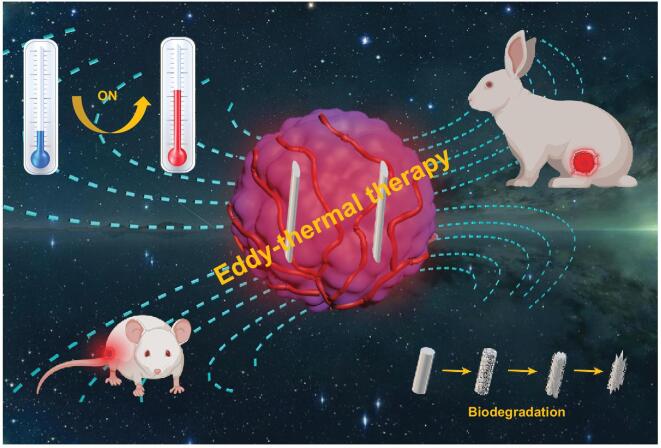
Schematic illustration of MHT based on the eddy thermal effect of the biodegradable MgA rods.

Magnesium alloy (MgA) with low elastic modulus, high mechanical compatibility, and excellent *in vivo* biocompatibility and biodegradability, has been extensively used in the clinic as an implant metal [33–36]. MgA has already been used in numerous patients for implantable medical devices, especially in the areas of orthopedics [37] (bone plates and screws, porous scaffold of bone tissue) and cardiovascular disease [38] (intravascular stent, vascular suture) [39–41]. Herein, we propose that biodegradable MgA with a strong eddy thermal effect could be used for magnetic hyperthermal ablation of tumors under an AMF with a low field intensity (Scheme 1). It was found that MgA rods under an AMF showed obvious length-dependent and diameter-dependent temperature increases originating from the eddy thermal effect. According to the heating range calculation, a MgA rod (D = 0.7 mm, L = 4 mm) could lead to the thermal ablation of a tumor mass no smaller than 3 mm × 7 mm under an AMF with a low field intensity (H_appl_ × f_appl_ = 2.0 × 10^9^ A · m^−1^ · s^−1^), so as to realize effective and accurate magnetic hyperthermia ablation of tumors and minimize the damage to normal tissues. *In vitro* experiments demonstrated a significant cell-killing effect owing to the AMF-induced heating of MgA added into the cell culture. *In vivo* MHT experiments were further carried out by inserting MgA rods into mouse tumors as well as into much larger-sized rabbit tumors, achieving excellent tumor ablation effects by placing mice or rabbits into AMFs with the field intensities at H_appl_ × f_appl_ = 2.0 × 10^9^ A · m^−1^ · s^−1^ and H_appl_ × f_appl_ = 1.5 × 10^9^ A · m^−1^ · s^−1^, respectively. Moreover, we found that MgA rods showed excellent biocompatibility and ∼20% of their mass would be degraded within three months after *in vivo* implantation. Thus for the first time our work presented biodegradable MgA macroscale rods as an eddy thermal magnetocaloric agent to ablate tumors under a low-field-intensity AMF.

## RESULTS AND DISCUSSION

**Figure 1.**
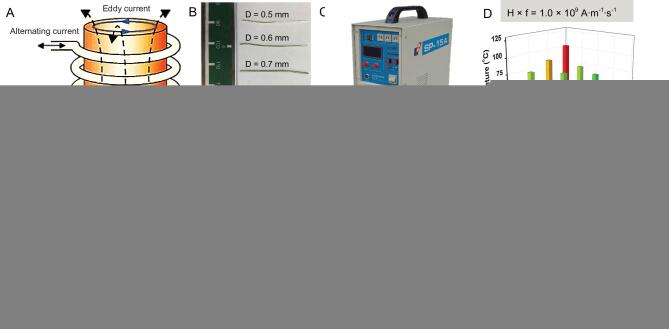
AMF-induced eddy thermal effect of MgA rods. (A) Schematic illustration to show AMF-induced eddy thermal heating of MgA rods. Photograph of MgA rods with different diameters (D: 0.50 ± 0.01, 0.60 ± 0.01, 0.70 ± 0.01, 0.80 ± 0.01 and 0.90 ± 0.01 mm) (B) and the high-frequency AMF induction equipment (C). (D–H) The temperature increase data of MgA rods with different lengths (2.0 ± 0.1, 4.0 ± 0.1, 6.0 ± 0.1 and 8.0 ± 0.1 mm) or different diameters (0.50 ± 0.01, 0.60 ± 0.01, 0.70 ± 0.01, 0.80 ± 0.01 and 0.90 ± 0.01 mm) under AMF with different field intensities.

According to Faraday's law of electromagnetic induction, when an alternating current is applied in the coil, an AMF will be generated inside the coil. The AMF will further produce an alternating electric field, namely the eddy current electric field. The eddy electric field forms an eddy current in the conductor, which causes a thermal effect in the conductor, namely an eddy thermal effect (Fig. 1A). The macroscopic metal materials can generate heat by eddy current under the AMF, and the heat generation efficiencies would be affected by the metal size and AMF field intensities. Moreover, as the volume of the conductor increases, the eddy current effect becomes more significant, and the thermal effect becomes more obvious [32].

To investigate the eddy thermal effect of MgA, a kind of commercial MgA with the composition of Mg:Zn:Ca (97.7:2.0:0.3) was used. The heating properties of MgA rods (Fig. 1B) with different lengths (L: 2, 4, 6, and 8 mm) and diameters (D: 0.5, 0.6, 0.7, 0.8 and 0.9 mm) under AMFs with different field intensities (H_appl_ × f_appl_ = M × 10^9^ A · m^−1^ · s^−1^, M = 1.0, 1.5, 2.0, 2.5 and 3.0) were carefully investigated (Fig. 1C–H; Fig. S1, online supplementary data). Notably, obvious length-dependent and diameter-dependent temperature increases of MgA rods were observed under the different field intensities for 120 s. As expected, stronger field intensities of AMF resulted in stronger eddy thermal heating of the MgA rods. Interestingly, with prolonged MgA rod lengths and enlarged MgA rod diameters, the AMF-induced heating would be significantly increased as evidenced by the temperature increase data. Additionally, it was found that the diameter change had more influence on the heating performance than the length change. When the length of MgA changed from 2 mm to 8 mm (D = 0.5 mm, H_appl_ × f_appl_ = 2.0 × 10^9^ A · m^−1^ · s^−1^), the temperature increased by ∼10°C (from 41.2 to 51.9°C). However, the temperature increased by more than 45°C (from 41.2 to 87.7°C) when the diameter changed from 0.5 to 0.9 mm (L = 2 mm, H_appl_ × f_appl_ = 2.0 × 10^9^ A · m^−1^ · s^−1^), probably due to the smaller specific surface area leading to a more significant eddy thermal effect. Notably, among three major types of heat transfer mechanisms, heat conduction, heat convection, and heat radiation, the heat conduction and heat convection are positively correlated with temperature difference, and the thermal radiation is positively correlated with the surface area and temperature difference [42–44]. In our system, owing to the rapid heating of MgA rods under the AMF, MgA rods with larger sizes exhibited enhanced heating performance due to their stronger eddy thermal effect, which is size-dependent, despite higher heat loss for the larger rods.

Furthermore, we compared the AMF-triggered induction heating performance of MgA rods with traditional magnetocaloric agents such as pure iron NPs and iron oxide (Fe_3_O_4_) NPs under the same AMF intensity (H_appl_ × f_appl_ = 3.0 × 10^9^ A · m^−1^ · s^−1^), by adding the same weight amount of each sample (10 mg) to 1 mL water (Fig. S2, online supplementary data). Based on the measured water temperature, we found that the induction heating effect of MgA rods was much stronger than that of magnetic NPs. Moreover, MgA rods with larger diameters showed a more significant eddy thermal effect.

**Figure 2.**
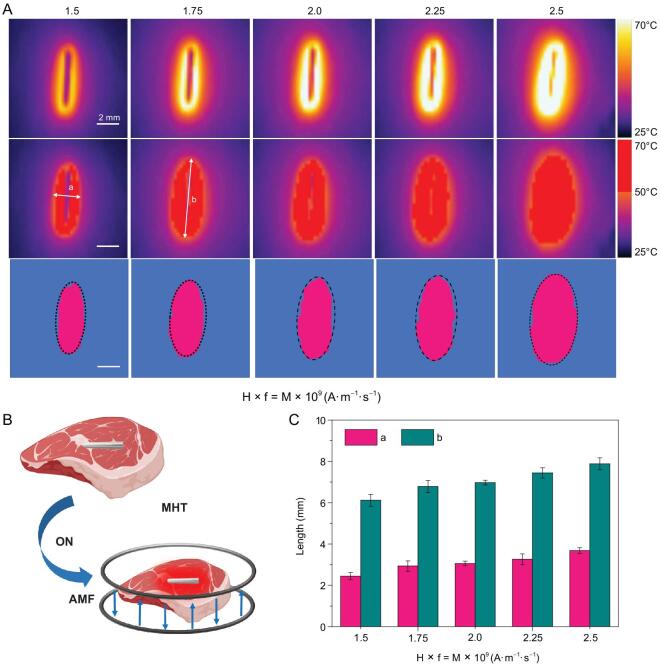
Effective killing ranges based on heating of pork tissues with MgA rods under AMFs. (A) Thermal imaging of MgA rod (D = 0.7 mm, L = 4.0 mm) under AMF with different field intensities (H_appl_ × f_appl_ = M × 10^9^ A · m^−1^ · s^−1^; M = 1.5, 1.75, 2.0, 2.25 and 2.5). (B) Schematic illustration of the effective killing range simulation. (C) The effective killing range of MgA rods under AMF with different field intensities based on the heat diffusion ranges in pork tissues with the temperature above 50°C. a: length; b: width.

In the process of hyperthermia, it is necessary to study the effective killing range of MgA rods under an AMF for killing the tumor with maximum efficiency while reducing trauma to normal tissues. However, the different heat conduction media would affect the range of temperature distribution. To better simulate the MHT-induced effective killing range, we took MgA rods (D = 0.7 mm, L = 4 mm) as the heat source and pork tissue (2 cm × 2 cm) as the heat conduction medium to carefully investigate the effective killing range, which was defined as the heat diffusion range to the area above 50°C (Fig. 2A and B). Considering the heat exchange effect, the temperature diffusion situation basically reached a steady state within 10 min. Therefore, the effective killing range was analyzed by using the temperature diffusion situation at that time point. As the field intensities increase, the effective kill ranges (with temperatures ≥ 50°C) showed an obvious increase (Fig. 2C). As a result, a MgA rod (D = 0.7 mm, L = 4 mm) could lead to the full ablation of a tissue mass with a size no smaller than ∼3 mm × 7 mm under a low-field-intensity AMF (H_appl_ × f_appl_ = 2.0 × 10^9^ A · m^−1^ · s^−1^). Therefore, based on the above information, we would be able to plan the MHT experiments to achieve accurate tumor hyperthermia ablation by selecting the appropriate number of MgA rods and how they are inserted into tumors before the AMF treatment.

**Figure 3.**
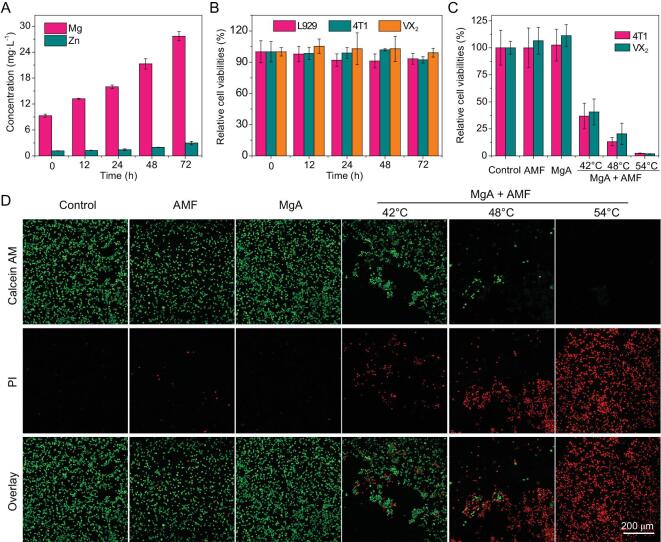
*In vitro* MHT with MgA rods. (A) Ion concentrations in RPMI cell culture medium immersed with MgA rods with different periods of time. (B) The viabilities of L929 cells, 4T1 cells and VX_2_ cells after 24 h of exposure to MgA-treated cell culture medium. (C) Relative viabilities of 4T1 cells and VX_2_ cells after MHT treated by MgA rods. (D) Confocal fluorescence images of VX_2_ cells stained with Calcein AM (AM, green, live cells) and propidium iodide (PI, red, dead cells) after various treatments. The temperature of treatment was controlled at ∼42°C (H_appl_ × f_appl_ = 2.0 × 10^9^ A · m^−1^ · s^−1^), ∼48°C (H_appl_ × f_appl_ = 2.5 × 10^9^ A · m^−1^ · s^−1^) and ∼54°C (H_appl_ × f_appl_ = 3.0 × 10^9^ A · m^−1^ · s^−1^), respectively.

To study the cytotoxicity of MgA, a RPMI (Roswell Park Memorial Institute) cell culture medium was used to incubate with the sterilized MgA rods for different periods of time (0, 12, 24, 48 and 72 h). Notably, the ion contents of Mg and Zn in the medium slightly increased, probably due to the slow degradation of MgA with the increase of time (Fig. 3A). The maximum ion content of Mg in the reaction medium was about 28 mg L^−1^ after soaking for 72 h as measured by inductively coupled plasma optical emission spectrometry (ICP-OES), indicating that MgA rods gradually degraded in the physiological solution. In order to test the potential toxicity of etched ions from MgA, L929 murine fibroblast cells, 4T1 murine breast cancer cells, and VX_2_ rabbit hepatocellular carcinoma cells were incubated with the culture medium, which was pre-soaked with MgA rods for 72 h. After 24 h, the standard methyl thiazolyl tetrazolium (MTT) cell viability assay was conducted, revealing no significant cytotoxicity of those ions etched from MgA rods to the cells (Fig. 3B).

To test the *in vitro* cell-killing efficacy of MgA under an AMF, MgA rods (D = 0.7 mm, L = 4 mm) were added to the 12-well cell culture plate pre-cultured with 4T1 cells or VX_2_ cells. The cells co-incubated with the MgA rods were then exposed to an AMF for 5 min. By adjusting the field intensity of the AMF, the temperature was controlled at ∼42°C (H_appl_ × f_appl_ = 2.0 × 10^9^ A · m^−1^ · s^−1^), ∼48°C (H_appl_ × f_appl_ = 2.5 × 10^9^ A · m^−1^ · s^−1^) and ∼54°C (H_appl_ × f_appl_ = 3.0 × 10^9^ A · m^−1^ · s^−1^), respectively. More than ∼75% of cells were killed at temperatures higher than 48°C, and nearly 100% of 4T1 cells and VX_2_ cells were damaged at a temperature of 54°C (Fig. 3C). Moreover, the Calcein AM (AM, live cells) and propidium iodide (PI, dead cells) co-staining were also performed to determine the killing effect of MgA rods under an AMF for VX_2_ cells (Fig. 3D). Similar to the MTT results, no noticeable red fluorescent signals corresponding to dead cells were observed in the samples treated with MgA alone or AMF treatment alone, while the vast majority of cells were necrotized (red fluorescence, positive in PI) for the sample incubated with MgA and exposed to AMF treatment at a temperature of 54°C.

**Figure 4.**
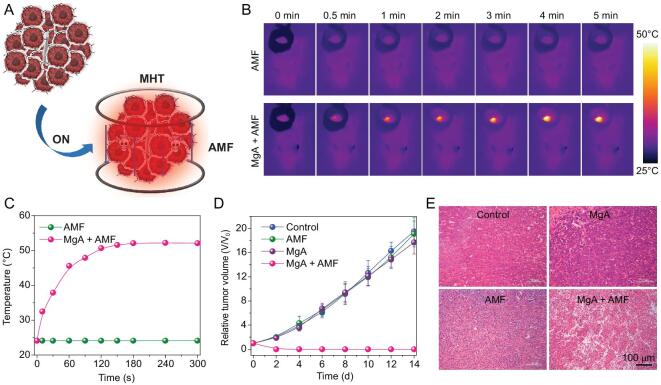
*In vivo* MHT treatment of 4T1 mouse tumors with MgA rods. (A) Schematic illustration of MHT with MgA rods to ablate tumors in mice. Infrared thermal images (B) and temperature change curves (C) of tumors in mice with or without implantation of MgA rods under AMF. (D) The growth curves of tumors after various treatments. (E) H&E stained tumor slices collected from different treatment groups.

Encouraged by the excellent eddy thermal performance of MgA rods, further systematic *in vivo* tumoricidal experiments were carried out. When tumor volumes reached ∼100 mm^3^, the 4T1 tumor-bearing mice were randomly divided into four groups: (i) PBS; (ii) MgA rods implantation; (iii) AMF only; (iv) MgA rods implantation + AMF. According to the heating range calculation, a MgA rod (D = 0.7 mm, L = 4 mm) could lead to the thermal ablation of a tumor mass with a size of ∼3 mm × 7 mm. Therefore, two MgA rods (D = 0.7 mm, L = 4 mm) were implanted into each tumor (∼6 mm × 6 mm) in our experiments (Fig. S3, online supplementary data). Those mice were anesthetized and then exposed to the AMF for 10 min with the field intensity at H_appl_ × f_appl_ = 2.0 × 10^9^ A · m^−1^ · s^−1^, which was much lower than those reported field intensities for *in vivo* MHT using magnetic NPs (H_appl_ × f_appl_ > 5 × 10^9^ A · m^−1^ · s^−1^) (Fig. 4A) [26,27]. During AMF exposure, the temperature change was monitored by an infrared thermal camera (Fotric 225). Noticeably, the temperature of MgA-implanted tumors rapidly increased to 52.5°C under AMF, while no obvious temperature increase was observed in the control group under the same AMF treatment (Fig. 4B and C). Tumor volumes and body weights were measured every two days by using a digital caliper (Fig. 4D; Figs S4 and S5, online supplementary data). The results showed that the tumors in non-implanted mice with or without AMF exposure and MgA-implanted tumors without AMF exposure maintained rapid growth after the treatment. However, the MgA-implanted tumors with AMF exposure could be effectively eliminated, due to the accurate and effective AMF-induced thermal heating by MgA rods via the eddy thermal effect. Meanwhile, there was no significant body weight loss during the therapeutic process, suggesting the biocompatibility of the MgA rods. The high efficacy of treatment was also analyzed by the hematoxylin and eosin (H&E) staining of tumor slices post various treatments (Fig. 4E). Severe damage was observed in tumors owing to magnetic thermal heating with MgA rods, but not in other control groups.

**Figure 5.**
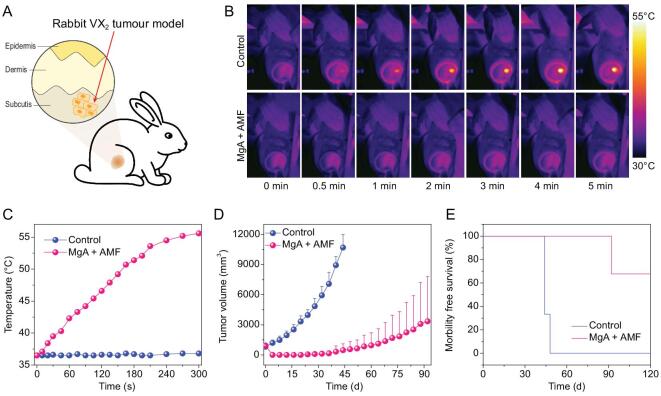
*In vivo* MHT treatment of VX_2_ rabbit tumors with MgA rods. (A) Schematic illustration of rabbit VX_2_ tumor model. Infrared thermal images (B) and temperature change curves (C) of tumors in rabbits with or without implantation of MgA rods under AMF. (D) The growth curves of tumors after various treatments. (E) Survival rates of tumor-bearing rabbits post various treatments. The tumor in one out of three rabbits was not completely eliminated by MgA-based MHT and later showed re-growth.

To demonstrate the advantages of MHT in deep tissue penetration, we further used this strategy to treat tumors of much larger sizes growing in rabbits. In our experiments, rabbits bearing VX_2_ liver tumors were randomly divided into two groups (n = 3); one group was implanted with MgA rods and the other group without implantation acted as control (Fig. 5A). Due to the large tumor size (∼800 mm^3^) before treatment, three MgA rods with larger diameter and longer length (D = 1.0 mm, L = 8.0 mm) were used for the rabbit experiments (Fig. S3, online supplementary data). As revealed by thermal imaging (Fig. 5B and C), the significant thermal heating effect of MgA rods was found under the lower field intensity (H_appl_ × f_appl_ = 1.5 × 10^9^ A · m^−1^ · s^−1^) owing to the eddy thermal effect, which resulted in the rapid increase of tumor temperature to ∼55°C. In contrast, no temperature increase was found in rabbit tumors without implantation of MgA under the same AMF treatment. The tumor volumes were measured every four days afterwards (Fig. 5D; Fig. S6, online supplementary data). While tumors without MgA implantation showed rapid growth after AMF treatment, MgA-implanted tumors were effectively ablated by AMF. In fact, tumors in two out of three rabbits in this group were completely eliminated without re-growth, and these two rabbits survived for over 90 days after MTH treatment with MgA (Fig. 5E). Therefore, the effective and accurate AMF-triggered tumor ablation could be used to treat relatively large-sized tumors.

**Figure 6.**
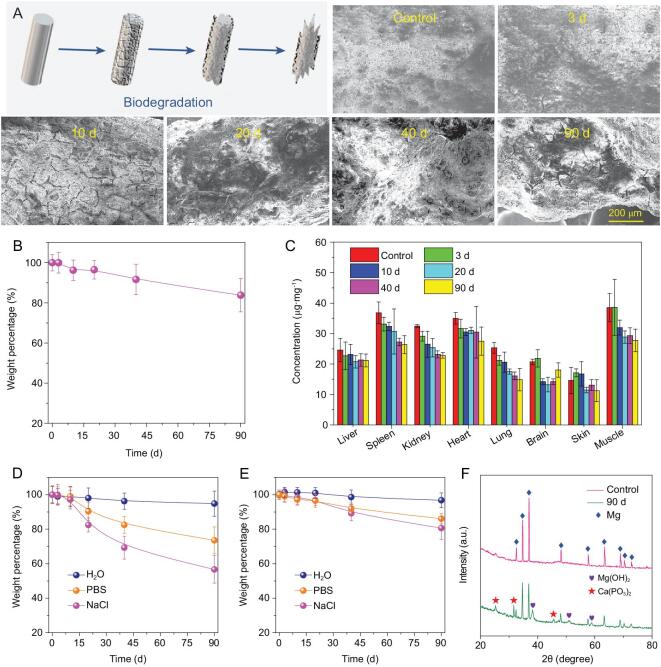
Biodegradation behaviors of MgA rods. (A) Scanning electron microscope (SEM) images to show the surfaces on MgA rods after *in vivo* implantation into mice for different periods of time. (B) The time-dependent mass loss of MgA rods after *in vivo* implantation into mice. (C) The Mg levels in major organs of mice after being implanted with MgA rods for different numbers of days. The Mg contents were measured by ICP-OES. No notable increase of Mg^2+^ levels was found in organs of implanted mice compared to the blank control. (D, E) The mass loss of MgA rods of two different dimensions (D: 0.7 mm × 4 mm; E: 1.0 mm × 4 mm) after being immersed in different solutions for different periods of time. (F) The XRD patterns of MgA after being immersed in PBS for 90 days.

Next, we carefully evaluated the biocompatibility and biodegradability of MgA used in our experiments. Healthy female mice were subcutaneously implanted with MgA rods (D = 0.7 mm, L = 4 mm, two MgA rods per mouse), which were taken out at different time points for examination. With the time increase, a large number of corrosion cracks appeared on the surface of the MgA rods, indicating the gradual *in vivo* degradation of MgA (Fig. 6A). Notably, we observed time-dependent weight losses for the implanted MgA rods, which showed a weight loss of ∼20% after three months (Fig. 6B). The induction heating decreased slightly with the degradation of MgA rods due to the decrease of the eddy thermal effect caused by the volume reduction (Fig. S7, online supplementary data). When measuring Mg levels in different organs of the mice, there was no significant increase of Mg levels in the main organs determined at different time points after MgA was implanted into the body, indicating that Mg^2+^ generated from the degraded MgA would show little retention in mouse organs (Fig. 6C). Hematological and histological examinations were then conducted to evaluate the potential toxic effect of the implanted MgA rods to the mice. All of the serum biochemistry indexes and hematology assay data were found to be normal in the MgA-treated groups compared with the control (Fig. S8, online supplementary data). Meanwhile, the histology examination of main organs further confirmed that implantation of MgA rods caused no appreciable side effects in the treated mice (Fig. S9, online supplementary data).

At last, we ought to understand the degradation chemistry of MgA. MgA rods were soaked in different aqueous solutions including water, saline (0.9% NaCl), or phosphate-buffered saline (PBS, containing 0.9 NaCl). It was found that the degradation rate of MgA in saline was higher than that in water, indicating that the increased ionic composition would accelerate degradation of MgA (Fig. 6D and E; Fig. S10, online supplementary data). It is generally believed that the presence of Cl^−^ could facilitate the degradation of metal such as MgA in this case, and the following reaction may occur: Mg + 2H_2_O = Mg(OH)_2_ + H_2_, Mg(OH)_2_ + 2Cl^−^ = MgCl_2_ + 2OH^−^ [45]. However, the degradation rate of MgA in PBS was lower than that in saline, probably because insoluble magnesium phosphate may be generated and deposited on the surface of MgA once it has been soaked in PBS, delaying the continued corrosion of the metal. With the increase of rod diameter, the degradation rate decreased due to the reduced specific surface area. To identify the product formed on the surface of MgA rods after three months of soaking in PBS, the XRD analysis showed that the peaks emerging from the tested sample corresponded to magnesium hydroxide and calcium metaphosphate, both of which should be non-toxic biocompatible components (Fig. 6F). All the above results illustrated that the biocompatible MgA rods could be gradually degraded after *in vivo* implantation.

## CONCLUSION

In summary, the non-magnetic biodegradable MgA was proven to be an efficient magnetocaloric agent for the thermal ablation of tumors under low-intensity AMFs. The AMF-induced heating effect of MgA rods that originated from the eddy thermal effect appeared to be dependent on the rod lengths and diameters. The effective killing range estimated by measuring the heat diffusion range within tissues with a temperature over 50°C could be used to design the magnetic hyperthermia treatment plan using MgA rods for effective and accurate tumor ablation. Such AMF-induced eddy thermal heating of MgA could be employed for *in vivo* ablation of tumors, not only to eliminate subcutaneous mouse tumors, but also for effective destruction of much larger-sized rabbit tumors. Moreover, the implanted MgA rods showed excellent biocompatibility and could be gradually degraded after *in vivo* implantation. This work not only broadens the application of MgA in biomedicine, but also provides a new strategy for accurate and effective tumor treatment under a low-field-intensity AMF in a minimally invasive manner, applicable even for deep-set and large tumors. Considering the wide clinical use of implantable MgA devices, such a strategy should hold great promise in clinical translation.

## METHODS

### Materials

Magnesium alloy (MgA, Mg:Zn:Ca = 97.7:2.0:0.3 wt%) was obtained from JingJun Materials Technology Co. Ltd. (Suzhou, China). All chemicals were of analytical grade and used without further purification.

### Characterization

SEM imaging was carried out by Gemini 500 SEM. The chemical phase analysis was carried out by using a PANalytical X-ray diffractometer (XRD), which was equipped with CuKα radiation (λ = 0.15406 nm). The absolute Mg and Zn contents were determined by ICP-OES. The temperature and thermal images were detected and recorded using an infrared thermal camera (Fotric 225).

### The eddy thermal effect of MgA studies

The MgA rods with different lengths (L: 2.0 ± 0.1, 4.0 ± 0.1, 6 ± 0.1 and 8.0 ± 0.1 mm) and diameters (D: 0.50 ± 0.01, 0.60 ± 0.01, 0.70 ± 0.01, 0.80 ± 0.01 and 0.90 ± 0.01 mm) were prepared. The AMF was provided by high-frequency induction heating equipment (SP-15A, Shenzhen Shuangping Power Technology Co. Ltd.), which was equipped with two different heating coils (diameters: 2.0 cm and 4.0 cm). In order to fix the MgA rods, a glass slide was placed in the gap of the heating coil, and the Mg alloy rod was put on the center of the horizontal glass slide.

During the measurement, each MgA rod was placed at the center of the heating coil, and the temperature of the MgA rod was monitored using an infrared thermal camera (Fotric 225). All the thermal imaging photographs were obtained by Fotric 225 and analyzed by Analyz IR software.

### *In vitro* toxicity studies

4T1 murine breast cancer cells, L929 murine fibroblast cells and VX_2_ rabbit hepatocellular carcinoma cells were purchased from the American Type Culture Collection (ATCC) and cultured in the standard cell culture medium at 37°C under 5% CO_2_ atmosphere. To demonstrate its cytotoxicity by the indication of cell viabilities, two MgA rods (D = 0.7 mm, L = 4.0 mm) were added into the cell culture medium for 12, 24, 48 or 72 h. The ion concentrations of Mg and Zn in the cell culture medium were measured by ICP-OES. The mixtures were separated out using a filter (pore size: 0.22 μm), and the remaining solution was used to culture cells.

For *in vitro* MHT, 4T1 cells or VX_2_ cells were incubated with two MgA rods (D = 0.7 mm, L = 4.0 mm) and exposed to the AMF for 5 min. The cell viabilities were determined by the MTT assay. For fluorescence imaging, treated VX_2_ cells were stained with Calcein AM (AM, living cell) and propidium iodide (PI, dead cell). All fluorescent images were acquired by confocal laser scanning microscope (CLSM, Zeiss Axio-Imager LSM-800).

### Animals and tumor xenograft models

Balb/c mice, nude mice and SPF New Zealand white rabbits were purchased from Nanjing Sikerui Biological Technology Co. Ltd., and all animal experiments were carried out following protocols approved by Soochow University Laboratory Animal Center. To set up the subcutaneous 4T1 breast tumor model, female balb/c mice (6 weeks) were subcutaneously inoculated in the back with 2 × 10^6^ 4T1 cells dispersed in ∼50 μL of PBS. To develop the rabbit tumor model, female nude mice (6 weeks) were subcutaneously inoculated with 2 × 10^6^ VX_2_ liver cancer cells dispersed in ∼50 μL of PBS. After the tumor grew to a size of about 1.0 cm × 1.0 cm, the mice were sacrificed to collect their tumors, which were cut into chunks (∼2 mm × 2 mm × 2 mm) and then implanted into the legs of each SPF New Zealand white rabbit.

### *In vivo* MHT

For *in vivo* MHT, 4T1-tumor-bearing mice were randomly divided into four groups (five mice per group) when the tumor volume reached ∼100 mm^3^: (i) PBS; (ii) MgA rods implantation; (iii) AMF only; (iv) MgA rods implantation + AMF. For mice in groups 2 and 4, tumors were implanted with MgA rods (D = 0.7 mm, L = 4.0 mm), with two rods for each tumor. The tumor sizes and body weights were measured every two days. The tumor volumes were calculated by the formula: volume = length × width^2^/2. At day 14, the mice were sacrificed and their tumors were collected, photographed, washed with saline and fixed in 4% neutral-buffered formalin. For the H&E staining, the formalin-fixed tumors were embedded in paraffin blocks and visualized by fluorescence optical microscope (DM4000M, Laica, Germany). For the rabbit tumor model experiments, VX_2_ tumor-bearing rabbits were randomly divided into two groups (three rabbits per group): (i) control; (ii) MgA rods implantation + AMF (H_appl_ × f_appl_ = 1.5 × 10^9^ A · m^−1^ · s^−1^). For group 2, the rabbits were implanted with three MgA rods (D = 1.0 mm, L = 8.0 mm), and the tumor size was measured every four days.

### Biocompatibility and biodegradability study

Healthy female balb/c mice were implanted with MgA rods (two rods per mouse, D = 0.7 mm, L = 4.0 mm) subcutaneously. At different time points, the MgA rods were taken out, placed in a solution of chromic acid for 1 min, and then rinsed repeatedly with deionized water. Finally, the MgA rods were put into an oven to dry before being weighed.

Major tissues from brain, liver, spleen, heart, lung and kidney of each group were dissected at different time points and cut into two halves. One half of the organs were fixed in 4% neutral buffered formalin, embedded into paraffin, sectioned by routine procedures for further H&E staining, and finally observed by fluorescence optical microscope (DM4000M, Laica, Germany). The other half of the organs and tissues were weighed, solubilized in aqua regia and measured by ICP-OES to determine Mg levels in those organs. Meanwhile, the blood samples were collected for blood biochemistry and hematology tests, which were conducted by Servicebio Biotechnology Co., Ltd. (Wuhan).

## Supplementary Material

nwaa122_Supplemental_File
