# Supplementary material for: Biodegradable magnesium alloy with eddy thermal effect for effective and accurate magnetic hyperthermia ablation of tumors
Source: Natl Sci Rev. 2020 Jun 10;8(1):nwaa122. doi: 10.1093/nsr/nwaa122 (PMC8288380; doi:10.1093/nsr/nwaa122)
Supplement: nwaa122_Supplemental_File [file nwaa122_supplemental_file.docx]

**Supporting Information**

**Biodegradable Magnesium Alloy with Eddy-thermal Effect for Effective and Accurate Magnetic Hyperthermia Ablation of Tumors**

Nailin Yang^1^, Fei Gong^1^, Liang Cheng^1^*, Huali Lei^1^, Wei Li^2^, Zongbin Sun^3^, Caifang Ni^2^,

Zhanhui Wang^3^, and Zhuang Liu^1^*

^1^Institute of Functional Nano & Soft Materials (FUNSOM), Jiangsu Key Laboratory for Carbon-Based Functional Materials and Devices, Soochow University, Suzhou 215123, China.

^2^Department of Vascular Surgery and Interventional Radiology, First Affiliated Hospital of Soochow University, No.188 Shizi St., Suzhou 215006, China.

^3^Luoyang Central Hospital affiliated to Zhengzhou University, Luoyang, China, 471000.

∗Corresponding authors. Email: [zliu@suda.edu.cn](mailto:zliu@suda.edu.cn);[lcheng2@suda.edu.cn](mailto:lcheng2@suda.edu.cn)

**Figure S1.** The heating curves of MgA rods with different length (2, 4, 6, and 8 mm), diameter (0.5, 0.6, 0.7, 0.8, and 0.9 mm) under AMF with different field intensities (H_appl_ × f_appl_ = M ×10^9^·A·m^-1^·s^-1^, M=1.0, 1.5, 2.0, 2.5, and 3.0).

**Figure S2.** The temperature change curves (A), temperature increase (B), and thermal IR images (C) of Fe NPs, Fe_3_O_4_ NPs, and MgA rods with different diameters under same AMF intensity. 10 mg of each sample was added into 1 mL water and placed into the magnetic coil for heating.

**Figure S3.** The position of MgA rods in the tumors of mice (A) or rabbits (B) when treated with AMF.

**Figure S4.** The body weight variation of 4T1 tumor-bearing mice post various treatments.

**Figure S5.** The representative photographs of 4T1 tumors collected from mice with various treatments.

**Figure S6.** Individual growth curves of VX_2_ tumors after various treatments.

**Figure S7.** Variation in the induction heating with the degradation of MgA rods (D = 0.7 mm, L = 4.0 mm).

**Figure S8.** The blood biochemistry and hematology data of mice implanted with MgA rods (D = 0.7 mm, L = 4.0 mm, two rods per mouse) at various time points.

**Figure S9.** The hematoxylin and eosin (H&E) staining of mice major organs (brain, heart, liver, spleen, lung, and kidney) to examine the histological changes after implantation of MgA rods in mice at different days.

**Figure S10.** The macroscopic change of MgA rods (1.0 mm × 4 mm, 0.7 mm × 4 mm) after immersion in different solutions with various periods of time.
